# Accreditation as a quality-improving policy tool: family planning, maternal health, and child health in Egypt

**DOI:** 10.1007/s10198-020-01240-6

**Published:** 2020-11-20

**Authors:** Amira El-Shal, Patricia Cubi-Molla, Mireia Jofre-Bonet

**Affiliations:** 1 Department of Economics, Faculty of Economics and Political Science, Cairo University, Giza, Egypt; 2 Office of Health Economics, London, UK; 3 Department of Economics, City, University of London, London, UK

**Keywords:** Primary health care, Policy evaluation, Morbidity, Outpatient, Public health, Middle-income, Accreditation, Maternal health, Child health, I11, I12, I18

## Abstract

Accreditation of healthcare providers has been established in many high-income countries and some low- and middle-income countries as a tool to improve the quality of health care. However, the available evidence on the effectiveness of this approach is limited and of questionable quality, especially in low- and middle-income countries. We exploit the interventions introduced under Egypt’s health sector reform program between 2000 and 2014 to estimate the effect of health facility accreditation on family planning, maternal health, and child health outcomes. We use difference-in-differences fixed-effects and propensity score matching difference-in-differences models. To do so, we *spatially* link women to their nearest mapped health facilities using their global positioning system coordinates. We find that accreditation had multiple positive effects, especially on delivery care and child morbidity prevalence. The effects appear to weaken over time though. Our findings suggest that facility accreditation can be effective in improving family planning, antenatal care, delivery care, and child health, but stress the need to study how the effects can be sustained.

## Introduction

In 1997, Egypt launched a comprehensive Health Sector Reform Program (HSRP) to address fundamental challenges in the healthcare system. The HSRP introduced an integrated package of service delivery and financing interventions to address the means by which primary health care (PHC) is financed, delivered, organized, and managed [1, 2]. One of the cornerstones of the HSRP was the facility accreditation program, which was defined by the country’s Ministry of Health and Population (MOHP) as a process for evaluating PHC facilities according to a set of standards that define activities and structures that directly contribute to improved patient outcomes. The main aim of the program was to provide the HSRP with a framework for continuous quality improvement [3, 4].

Accreditation of healthcare providers has been established in many high-income countries and some low- and middle-income countries (LMICs) as an approach to improve the quality of care that combines the two elements of quality assurance and quality improvement [5, 6]. There is consistent evidence that accreditation programs improve the process of care in all settings, especially those related to the quality of healthcare services [7–11]. However, there is limited evidence on the effectiveness of accreditation on patient-related outcomes in all settings [12–14]. One interesting study on patient outcomes in a high-income country is Falstie-Jensen et al. [15], who report a statistically significant association between persistent low compliance with accreditation and higher 30-day mortality and longer length of stay at public Danish hospitals. However, Lam et al. [16] report that U.S. hospital accreditation is not associated with lower mortality and is only statistically weakly associated with lower re-admission rates for some medical conditions. Similarly, accreditation of public health hospitals in Saudi Arabia is found to have no significant effect on mortality rates but significant positive effects on infection and length of stay [17].

Although a number of studies explored the effect of accreditation on the quality of care in LMICs (e.g., [18]), we could only identify one study on the effect of quality improvement through accreditation on patient outcomes [19]. This study concludes that accreditation of Chinese chest pain centers was associated with better in-hospital outcomes for acute myocardial infarction patients. Since accreditation usually entails significant costs, investigating its effectiveness is crucial, especially in settings where resources are extremely constrained. Moreover, in light of evidence on the non-monotonic effect of access to health care on gender inequality [20], it is particularly important to estimate the effect of having access to an accredited facility on disadvantaged groups, such as women and children.

We attempt to fill the gap in the literature by investigating the effect of accreditation of PHC units on the quality of care and patient outcomes in Egypt, a middle-income country. The paper exploits the quasi-natural experiment associated with the introduction of Egypt’s facility accreditation program to estimate the effect of having access to an accredited facility on a set of family planning, maternal health, and child health outcomes. To do this, difference-in-differences (DiD) fixed-effects models are used for the period 1992–2014. DiD models are also combined with propensity score matching (PSM) for the specific periods of 2000–2005, 2005–2008, and 2008–2014.

The paper is organized as follows: section “Background” provides some background; section “Data” describes the exploited data; section “Estimation methods” explains our empirical strategy; section “Results” presents some descriptive statistics and estimation results; section “Discussion” discusses the paper’s findings; and section “Conclusion” concludes. Appendix contains descriptive statistics, robustness checks, and extensions.

## Background

### The facility accreditation program

In 1997, the Government of Egypt (GOE) launched the HSRP, a new PHC strategy to reform the health system in phases over a period of 15–20 years. The program came into operation in 2000 [2]. The simultaneous implementation of the program across the country was deemed infeasible due to pre-existent constraints in the healthcare system and the complex nature of interventions to be introduced. Hence, GOE decided to implement the program over phases. The targeting took place at the district level in the participating governorates, whose master plans relied on a social vulnerability index to target districts of the most vulnerable populations [1]. Early entrants to the program included a group of PHC facilities in the governorates of Alexandria, Menoufia, and Sohag, which represent, respectively, urban governorates, Lower Egypt, and Upper Egypt. The three pilot governorates represent the three major regions in Egypt. Other governorates followed subsequently [1].

One of the key marketing points of the HSRP service provision reform of PHC was that it would improve access to quality care. To ensure this, the facility accreditation program was established by MOHP as a process to monitor and facilitate the quality of services and influence the behavior of healthcare providers. Thus, as part of the HSRP, accreditation became obligatory to all PHC facilities in the districts targeted by GOE to join the program. Technical assistance was provided to develop improvement plans [3].

In the preparatory phase of the facility accreditation program, a PHC facility needed to meet the following criteria: (i) had a process to monitor, evaluate, and improve quality of care; (ii) had a patient record system; (iii) provided a defined package of services including reproductive health obstetrics and gynecology, neonatal care, pediatric and adult medical care, basic emergency care, preventive health services, and ambulatory care; and (iv) had been in operation for at least six months, had appropriate license by MOHP and relevant medical union, and operated within the laws and regulations [21].

The survey was a key step in the facility accreditation program. A site visit to PHC facilities was conducted by a team of experts trained in accreditation using pre-set accreditation survey instruments and tools. The purpose of the accreditation survey was to evaluate the extent to which a facility complied with the nationally established accreditation standards, and accordingly, determine whether it was to be awarded or denied accreditation. The assessment initially included eight categories: patient rights, patient care, safety, management of support services, management of information, quality improvement program, family practice, and management of the facility. Optimal standards in each category focused on key processes, activities, or outcomes that facilities should achieve [2, 3, 22].

During the accreditation survey, trained surveyors used three approaches to collect data and measure compliance with the established standards: review of specific administrative and clinical records; observe the performance of specified tasks in particular areas; and conduct personal interviews. If a facility scored 80% or above in the total survey scores, it was granted full accreditation for a period of 2 years. If a facility scored between 50 and 79% of the total survey score, it was granted provisional accreditation for one year, after which a re-assessment survey is conducted to investigate if the deficits pointed out by the first round have been addressed. If a facility scored less than 50%, accreditation was denied [22]. The scoring by areas is presented in Tables 3 and 4 in Appendix A: the facility accreditation program.

In terms of contribution to the total score, the quality dimension *patient care* contributed the most. This dimension measured the extent to which patients received appropriate care, and focused on compliance with clinical practice guidelines and appropriate diagnosis [22]. Details of the *patient care* assessment process are included in Appendix A: the facility accreditation program (see Box A.1).

### Anticipated effect of the facility accreditation program

One important characteristic of healthcare markets is the presence of asymmetric information [23]. It is well known that healthcare providers may act as ‘imperfect’ agents of patients and over or under provide care or fail to deliver the adequate health care quality, which can become a health concern. Different interventions, including accreditation of providers, have evolved in response to these problems. By subjecting healthcare providers to a formal process that makes them meet pre-determined standards, accreditation is expected to minimize variations in medical practice, eliminate medically inappropriate care, control costs, and address the possibility that quality is underprovided [24, 25]. Recent studies suggest that accreditation can influence quality of care through three mechanisms: coherence, organizational buy-in, and collective quality improvement action [26], with possible effects on patient-related outcomes. Earlier studies suggest that the accreditation process can stimulate organizational changes that enhance the quality of care (e.g., [27]).

In the context of this study, accreditation is expected to have a direct effect on some maternal health, child health, and family planning outcomes, through improving the quantity and quality of pertinent health services provided, and an indirect effect on other outcomes. Accreditation of health facilities certifies high compliance with standards defining activities and structures that directly contribute to improved patient outcomes. Hence, within the quality dimension *patient car*e, accreditation standards established to measure compliance of facilities in the sub-areas of antenatal care (ANC), integrated management of child illnesses (IMCI), and family planning are expected to have a primary effect on ANC coverage (number of visits), quality of ANC (being informed of signs of pregnancy complications, weight measurement, blood pressure measurement, and urine sample collection), child morbidity prevalence (acute respiratory infection (ARI), fever, and diarrhea), and informed choice of contraceptive methods (knowledge of side effects of contraceptive method used and knowledge of other methods of contraception that could be used). These outcomes reflect some of the standards assessed during the accreditation survey (see Appendix A: the facility accreditation program, Box A.1). We expect improvements in these outcomes in accredited compared to non-accredited facilities.

In parallel, accreditation is expected to have a secondary effect on the utilization of antenatal and delivery care services. Quality improvement in accredited facilities introduces an incentive for individuals to seek care at these facilities. The effect of this incentive is expected to be more significant in the sub-areas of care included in the assessment of the accreditation survey. Thus, we expect having access to an accredited facility to be associated with higher ANC coverage, higher institutional birth-delivery, and higher skilled assistance during delivery. This expectation holds given that accredited facilities were not functioning at full capacity prior to accreditation and can increase supply in the short term.

### Evidence on the effect of accreditation

There exists a large body of literature on the effects of accreditation as a quality signaling device for firms (for instance, a good summary on firm behavior and accreditation can be found in Dranove and Jin [28]). However, less is known about the impact of accreditation on healthcare provision and about patient health outcomes.

The majority of studies on accreditation in health care in LMICs report on its positive effects on process indicators. A recent study by Terra and Berssaneti [11] provides evidence that accreditation promoted healthcare processes in Brazilian hospitals, thus strengthening the culture for healthcare quality and increasing patients’ satisfaction. Similarly, in Brazil, Saut et al. [10] indicates that accreditation mainly influenced the internal processes of healthcare organizations. Saadati et al. [29] show that the accreditation of an Iranian teaching hospital improved patient-centeredness, patient safety, logistics and managerial processes, as well as decision-making.

Several studies in LMICs focus on accreditation’s effect on compliance with quality standards [30–33]. A quasi-experimental study in Egypt found that accredited non-governmental health units had higher compliance with quality standards compared with non-accredited units [30]. Another study in Egypt on public clinics found that providers that had earned an accreditive *Gold Star* were more likely to adhere to higher quality practices in counseling and examination than non-*Gold Star* facilities [32]. According to a descriptive study in Zambia, a national hospital accreditation program was associated with significant improvement in compliance of accredited hospitals with standards in overall scores and in seven out of 13 functional areas [31]. In South Africa, Salmon et al. [33] used a randomized control trial to investigate the effect of an accreditation program on public hospitals and found that the processes’ and outcomes’ average compliance of accredited hospitals improved significantly, while no significant improvement was observed in non-accredited hospitals.

Besides compliance with standards, the majority of the studies report on the effect of accreditation on quality of care measures. These are, for the most part, not patient health outcomes, but downstream process indicators [18, 30, 32–35]. Unlike compliance with standards, there is no conclusive evidence on the effect of accreditation on quality of care. While Avia and Hariyati [8], El-Jardali et al. [34], Hong et al. [32], Quimbo et al. [35], and Reisi et al. [18] report positive effects of accreditation on different indicators of quality of care, studies employing more robust study designs report mixed effects. These are Salmon et al. [33] and Al Tehewy et al. [30], which used a randomized controlled trial and a quasi-experimental design, respectively. In a study based on data from hospitals in South Africa, Salmon et al. [33] found limited or no effect of a randomized accreditation program on quality measures apart from increases in perception of quality among nurses. In Egypt, Al Tehewy et al. [30] found a positive effect of accreditation of non-governmental health units on patient satisfaction with respect to all areas of health service (cleanliness, waiting area, waiting time, and staff performance). As for provider satisfaction, the study found a positive effect on the overall satisfaction score, but no significant difference in the mean satisfaction score between accredited and non-accredited units with respect to social environment, administrative environment, and family health model.

In conclusion, the available evidence on the effectiveness of quality improvement through accreditation of healthcare providers is limited and of questionable quality but suggests that accreditation can improve the process of care provided by different providers. However, evidence is limited on the effectiveness of accreditation on patient outcomes. One study found that accreditation of Chinese chest pain centers was associated with better in-hospital outcomes for acute myocardial infarction patients [19]. We could not identify any study on the effect of accreditation on key patient outcomes such as family planning, maternal health, and child health outcomes.

## Data

To answer our research questions, we exploit the Egypt Demographic Health Survey (DHS). This survey consists of two questionnaires: one for households (HHs) and the other for ever-married women (EMW), and has a consistent response rate of over 99% for all waves.^1^

For this study in particular, we make use of all the relevant data made available by the Egypt DHS on family planning and maternal and child health. We expect these variables to be affected by the changes in quality standards, policies, and procedures, which is the focus of accreditation assessment.^2^

To construct our dependent variables at the facility level, we collapse the responses of 97,990 women over the period 1992–2014 at the facility level, drawing from six DHS waves: 1992, 1995, 2000, 2005, 2008, and 2014. To do so, we use the Global Positioning System (GPS) coordinates of both interviewed women and health facilities to link each woman to the nearest mapped facility for each wave of the Egypt DHS. The aim is to identify women who live in the catchment area of accredited facilities (treatment group) and those in the catchment area of non-accredited facilities (control group). All eligible PHC facilities across Egypt are used during the joining process. We are confident that women do not bypass their closest PHC facility because they are obliged by MOHP to use the facility in their catchment area.

The gradual uptake of the facility accreditation program by health facilities provides a quasi-natural experiment. Thus, for our main explanatory variable reflecting reform status, we draw on facility-level data from MOHP to categorize facilities as treated (accredited, either fully or provisionally) and non-treated (non-accredited). To ensure that *treatment* reflects only accreditation, we remove from the sample accredited health facilities that were subject to additional interventions under the HSRP such as performance-based financing (PBF) and introducing user fees.

### Dependent variables

Having linked each woman to a respective health facility, we are able calculate health outcomes at the facility level for each of the Egypt DHS waves and obtain a panel. By construction, our dependent variables are at the facility-level: informed choice of contraceptive methods (‘family planning’), ANC, delivery care, and child morbidity prevalence, which we describe in detail below.

#### Family planning

As part of the family planning sub-area of the patient quality of care dimension, the accreditation surveyor checks if the facility has a good information/education/communication (IEC) system. For this sub-area, we include two family planning outcomes capturing the effect of accreditation on choice of contraceptive methods by calculating the percentage of current users of selected contraceptive methods who were informed of the side effect of or problems associated to the method used.^3^ Those receiving information on the efficacy and side effect of contraceptives used tend to have higher continuation rates than those who do not [36]. We also calculate the percentage of current users of selected contraceptive methods who were informed of other methods of contraception that could be used.^4^ Informed choice emphasizes that women choose the method that best satisfies their personal and reproductive health needs based on a thorough understanding of other methods of contraception they could use.

#### ANC

As part of the ANC sub-area of the patient quality of care dimension, the surveyor checks if physical examination is performed for all patients. We include six ANC outcomes that capture the effect of accreditation on the quality of ANC. We calculate the percentages of mothers who received the following components of ANC: being informed of signs of pregnancy complications, weight measurement, blood pressure measurement, and urine sample collection.^5^ As part of the accreditation survey, the surveyor also checks if the number of ANC visits falls within the clinical guidelines. Therefore, we calculate an outcome of ANC coverage indicator (at least four visits).^6^ This outcome is used as a global preferred outcome of access to and use of health care during pregnancy to track performance in maternal health programs.^7^ A pregnant woman is expected to receive health interventions during antenatal visits that can be vital to her health and the health of her infant as well.

#### Delivery care

As part of the ANC sub-area of the patient quality of care dimension assessed by the accreditation survey, the surveyor assesses patient’s knowledge and understanding of delivery services provided in the facility. Egypt DHS data allows us to calculate two delivery care outcomes to capture the effect of compliance with the accreditation standards in this regard: institutional delivery^8^ and skilled assistance during delivery.^9^ The two outcomes are widely advocated for reducing maternal, perinatal, and neonatal mortality. Institutional delivery captures the effect of accreditation on expanding access to childbirth facilities and, more importantly, is a proxy measure of maternal and neonatal morbidity and mortality.^10^

The second but most important measure of delivery care included in our analyses is skilled assistance during delivery. Empirical literature provides evidence that wider access to professional care during pregnancy and childbirth reduces maternal mortality. Women assisted by skilled health personnel during delivery are less likely to die from any cause related to or aggravated by childbirth [37].

#### Child morbidity prevalence

As part of the IMCI sub-area of the *patient care* dimension of quality assessed by the accreditation survey, the surveyor checks if child is checked for cough, diarrhea, sore throat, ear infection, and fever. We use the prevalence of childhood ARI, fever, and diarrhea from the Egypt DHS data as three outcomes reflecting morbidity prevalence.^11^

ARI is the leading infectious cause of death in children worldwide.^12^ Diarrheal diseases are the second leading cause of death in children under age five [38]. The risk of under-five mortality can be diminished substantially through reducing the prevalence of ARI and diarrheal diseases and encouraging women to seek treatment for their children at a health facility and/or from a healthcare provider.^13^

### Explanatory variables

The explanatory variables included in the analyses of this study are a treatment variable that reflects participation in the facility accreditation program, facility-level controls, district-level social and economic controls, and regional dummies to control for regional variation.

In particular, the facility characteristics include labor force, the facility’s building condition, and population coverage. For labor force, we incorporate the number of eight types of workers in a health facility: practitioners, specialists, pharmacists, nurses, lab technicians, X-ray technicians, health observers, and social workers. For building infrastructure, a dummy variable that describes the condition of a facility building as ‘bad,’ ‘average,’ or ‘good’ is included. As for population, we control for the size of population in the facility catchment area. This information is obtained from Egypt’s MOHP.

At the district level, we use Egypt’s 2006 Population and Housing Census to construct eight district-level social and economic controls: illiteracy ratio, unemployment ratio, income dependency ratio, inaccessibility to electricity, inaccessibility to potable water, average family size, HH crowding factor, and population size. In addition, regional dummies are defined for fully urban governorates, urban Lower Egypt, rural Lower Egypt, urban Upper Egypt, rural Upper Egypt, and frontier governorates. These district-level covariates control for both the selection criteria of the HSRP targeting and the demographic variation across districts. The regional targeting of the HSRP followed a socio-economic vulnerability index that was constructed from the eight social and economic indicators outlined earlier.

## Estimation methods

### Difference-in-differences

As the accreditation policy is staggered, we first follow Wooldridge [39] and use a general DiD fixed-effects model to estimate the effect of accreditation by comparing the health outcomes of accredited facilities (treatment group) to that of non-accredited facilities (control group) before and after accreditation (treatment) between 1992 and 2014.

Accredited and non-accredited facilities presumably differ in observed characteristics, such as labor force, and unobserved ones too, such as managerial ability. The DiD method controls for both observed and unobserved characteristics that are time invariant. Fixed effects further eliminate any confounding that might be caused by facility effects, whether observed or unobserved, which are constant over time within each facility. With regard to time-varying unobservable factors, we report in Appendix C: checks and robustness extensions the results of the parallel-trends test, which provide evidence of the absence of unobserved time-varying confounding, establishing the unbiasedness of our DiD estimates.

Treatment self-selection is not a concern in our context as treatment, i.e., the decision of whether or not to obtain accreditation is exogenous. Accreditation is not a function of some choice of the treated unit, but is rather a function of a policy that differentially affects units based on pre-determined characteristics. As noted earlier, the facility accreditation program is only rolled out in certain geographical areas. Hence, whether one facility can obtain accreditation or not is neither self-selected nor screened.

For each health facility $i$ at time $t$, we estimate the following DiD fixed-effects model:

1 $$\it y_{it} = \alpha + \beta {policy}_{it} + \gamma {year}_{t} + \delta_{i} + \varepsilon_{it} ,$$

${y}_{it}$ denotes a health outcome of interest $y$ for facility $i$ at time $t$. We include outcomes of informed choice of contraceptive methods, ANC, delivery care, and child morbidity prevalence drawing from secondary data from Egypt DHS. The variable $\it {\mathrm{\it policy}}_{it}$ equals 1 if facility $i$ is subject to accreditation in year $t$. $\it {\mathrm{\it year}}_{t}$ is a time-period dummy. ${\delta }_{i}$ is the unobserved facility effect.

Additionally, to compare between the effects of accreditation for the specific periods of 2000 to 2005, 2005 to 2008, and 2008 to 2014; we estimate the typical DiD specification below for each health facility $i$ at time $t$.

2 $$\it \it y_{it} = \alpha + \beta acc_{it} + \gamma d_{{{post}}} + \delta acc_{it} \times d_{{{post}}} + \zeta fac_{i} + \eta dist_{i} + \varepsilon_{it} { },$$

where $t$ equals 0 for the baseline years (2000, 2005, or 2008) and 1 for the follow-up years (2005, 2008, or 2014); ${acc}_{it}$ is an indicator variable that takes value 1 if facility $i$ is accredited and 0 if not; $\it {d}_{\mathrm{\it post}}$ is an indicator variable for the follow-up year; the interaction term $\it {acc}_{it}\times {d}_{\mathrm{\it post}}$ measures the effect of accreditation in the follow-up year; and $\delta$, our main coefficient of interest, captures the effect of accreditation on the outcome at the facility level.

Finally, to eliminate potential unobserved heterogeneity and account for possible differences between accredited and non-accredited facilities prior to accreditation, the vector ${fac}_{i}$ contains facility-level controls that reflect different characteristics of facility $i$ including labor force, the facility’s building condition, and population coverage; and ${dist}_{i}$ is a vector of district-level controls including social, economic, and demographic characteristics of the district in which facility $i$ is located.

### Propensity score matching difference-in-differences

The targeting of the reform interventions at the district level under the HSRP followed a socio-economic vulnerability index of the areas around health facilities. As such, the comparison of health outcomes without accounting for this would be biased. To ensure that no bias exists due to targeting, we combine DiD with the PSM approach.^14^ Matching on observables mitigates the potential bias by pairing accredited and non-accredited health facilities based on pre-accreditation observable characteristics, which were initially used by GOE for accreditation targeting. Additionally, as a stand-alone method, DiD can be used to identify treatment effects if there is a selection based on (time-invariant) non-observables. Thus, while conventional PSM cannot account for non-observables, combining matching with DiD enables us to account for both the effect of observable and unobservable heterogeneity that is constant over time, as well as the targeting policy. To minimize any potential bias due to time-varying unobservable factors, we also control for an extensive set of facility-level characteristics and population coverage of facilities. Reassuringly, we generally find no significant differences in outcomes or characteristics between the population covered by treated and non-treated facilities (see Appendix C: checks and robustness extensions, Table 12). Table 12 suggests that our matching strongly satisfies the requirement of conditional independence.

To obtain the PSM DiD estimates, we follow Rosenbaum and Rubin [40]. We first apply PSM to match facilities and then extend the conventional DiD estimates by defining health outcomes conditional on propensity scores and applying semi-parametric methods to construct the differences. First, we match treated and control health facilities based on pre-treatment observable characteristics and use Kernel functions to assign weight to the *j*th control facility matched to the *i*th treated facility.^15^ As such, in our context, the propensity score is the probability of being targeted by the HSRP intervention given a set of observable social and economic indicators used to construct the socio-economic vulnerability index. Second, we estimate a DiD specification in Eq. (2) with health outcomes defined conditional on the propensity score generated earlier. The Kernel PSM DiD estimate for each treated facility *i* is calculated as

3 $$\hat{\delta }_{i} = \left( {y_{i{post}}^{T} - y_{i{pre}}^{T} } \right) - \mathop \sum \limits_{j \in C} \omega \left( {i,j} \right)\left( {y_{j{post}}^{C} - y_{j{pre}}^{C} } \right).$$

**Fig. 1:**
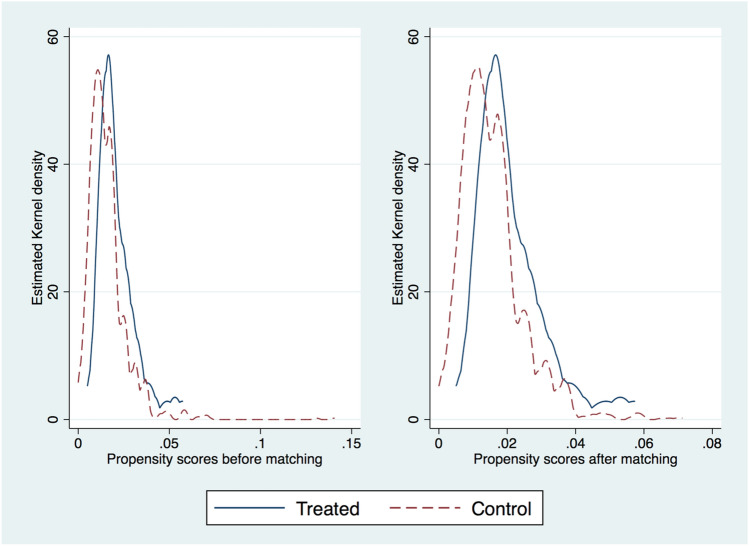
Estimated propensity scores: Kernel density estimates, 2000–2005

**Fig. 2:**
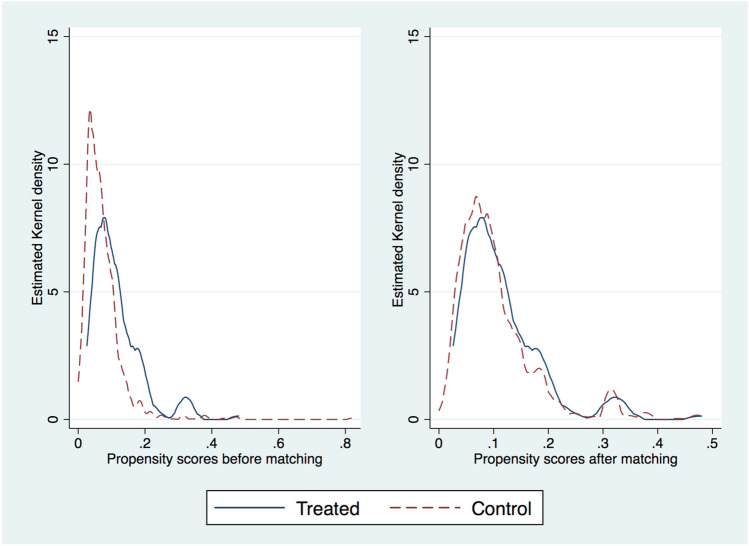
Estimated propensity scores: Kernel density estimates, 2005–2008

**Fig. 3:**
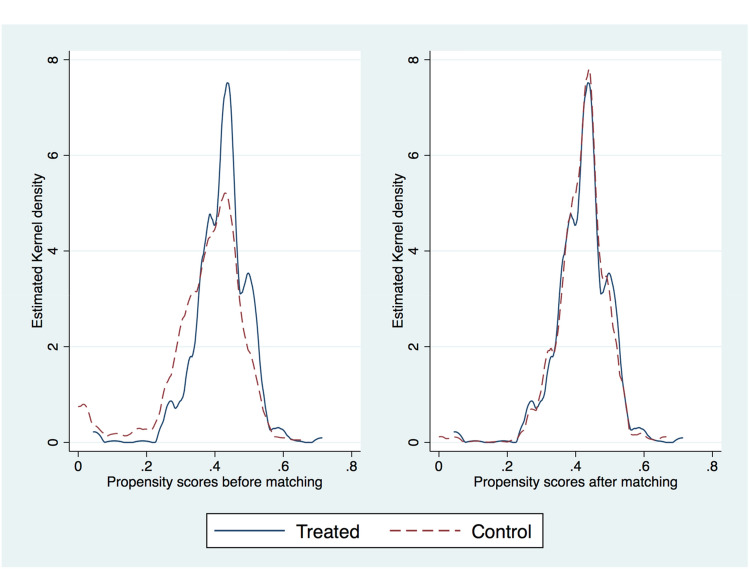
Estimated propensity scores: Kernel density estimates, 2008–2014

Prior to the DiD estimation, we verify that the *common support assumption* is satisfied by checking the overlap between treatment (accredited facilities) and control (non-accredited facilities) groups (see Appendix C: checks and robustness extensions, Figs. 1, 2, 3). Once the matching is applied, we use two-sample t-tests to examine if there are significant differences in the means of observable characteristics for both groups [40].

While we use district-level social and economic indicators to estimate the propensity score, facility-level characteristics are used as additional covariates later in the DiD estimations. For each of our health outcomes, we report the results for three study periods: 2000–2005, 2005–2008, and 2008–2014.

## Results

### Descriptive statistics

Table 7 in Appendix B: data sources and descriptive statistics summarizes the descriptive statistics of health outcomes and district characteristics of all health facilities observed between years 1992 and 2014. Overall, the table indicates relatively moderate variability across facilities and districts for most variables. The table shows that Egypt has an overall moderate level of ANC coverage. On average, 56% of pregnant women in Egypt reported at least four ANC visits. However, the country performed differently with respect to various ANC components. Women were more likely to be weighed (82%) and get their blood pressure measured (82%) during ANC visits but far less likely to be informed of signs of pregnancy complications (34%). In parallel, Egypt has a fairly high level of delivery care coverage through access to health facilities and skilled health personnel. On average, over 60% of women delivered their most recent birth in an institutional setting and over 70% of births were assisted by skilled health personnel. The country had a higher level of prevalence of childhood fever (23%) than ARI (14%) and diarrhea (13%).

The descriptive statistics of district characteristics of facilities based on which targeting took place are also reported in Table 8 in Appendix B: data sources and descriptive statistics. The table highlights the difference in the district characteristics between accredited and non-accredited facilities. We use the two-sample t-test to check whether the means of the two groups differ significantly. On average, districts to which accredited facilities belong have significantly higher HH overcrowding during the period 2000–2005; significantly lower illiteracy, income dependency, inaccessibility to electricity, and inaccessibility to potable water; smaller family size; and bigger population size during the period 2005–2008; and significantly lower income dependency, inaccessibility to electricity, and inaccessibility to potable water, smaller family size, and lower HH overcrowding during the period 2008–2014. These results suggest that the actual targeting of the HSRP did not strictly follow the socio-economic vulnerability index.

### Estimated effects of accreditation

Table 1 reports the DiD fixed-effects estimates of the effects of accreditation during the period 1992–2014. We additionally report the DiD and Kernel PSM DiD results for the specific periods 2000–2005, 2005–2008, and 2008–2014 in Table 2.

**Table 1:** Difference-in-differences fixed-effects estimates of the effects of accreditation, 1992–2014

	Family planning		ANC					Delivery care		Child morbidity prevalence		
	Knowledge of side effects	Knowledge of contraceptives	4 + visits	Informed of complications	Weight measurement	Blood pressure measurement	Urine sample collection	Institutional delivery	Skilled-assisted delivery	ARI	Fever	Diarrhea
Treat = 1	− 1.168 (2.357)	1.318 (2.461)	0.717 (1.823)	3.305 (2.729)	− 1.445 (1.780)	− 2.710 (1.788)	− 1.350 (2.501)	− 1.523 (1.781)	0.226 (1.647)	− 1.740 (1.506)	1.658 (1.820)	− 2.674** (1.197)
Years (Ref: 1992)												
1995	− 4.360** (1.990)		6.288*** (1.938)					7.678*** (2.007)	7.559*** (2.004)	14.132*** (1.286)	19.168*** (1.719)	3.028** (1.178)
2000	37.991*** (2.044)	40.478*** (1.855)	15.990*** (1.931)					21.227*** (1.904)	18.313*** (1.885)	0.398 (1.066)	− 3.950** (1.542)	− 5.935*** (1.090)
2005	38.417*** (1.960)	47.627*** (1.670)	36.737*** (1.786)	12.471*** (1.870)	25.150*** (1.577)	23.544*** (1.519)	26.875*** (1.800)	39.178*** (1.874)	33.367*** (1.843)	2.308** (1.122)	− 0.573 (1.580)	4.236*** (1.148)
2008	43.714*** (1.973)	51.344*** (1.841)	40.673*** (1.846)	15.131*** (2.039)	23.499*** (1.609)	23.435*** (1.550)	22.960*** (1.969)	42.058*** (1.910)	34.188*** (1.906)	2.688** (1.251)	− 7.025*** (1.653)	− 3.924*** (1.086)
2014	37.326*** (2.151)	51.250*** (1.950)	53.917*** (1.881)	24.522*** (2.139)	26.489*** (1.700)	31.460*** (1.634)	31.044*** (1.955)	54.279*** (1.992)	42.006*** (1.988)	6.863*** (1.368)	1.376 (1.756)	− 0.109 (1.257)
Constant	7.753*** (1.413)	5.522*** (1.071)	26.436*** (1.428)	19.129*** (1.226)	62.212*** (1.126)	62.062*** (1.100)	46.657*** (1.292)	30.473*** (1.523)	46.392*** (1.532)	9.287*** (0.860)	21.932*** (1.241)	13.829*** (0.853)
Obs	3526	3444	3808	2935	2937	2937	2937	3810	3810	3807	3807	3807

Each column represents a separate specification, i.e., the dependent variable is specified in the second row. Dependent variables are expressed in percentages. The observations are health facilities. Clustered standard errors are reported in parentheses

*, **, and *** denote statistical significance at the 10%, 5%, and 1% levels, respectively

**Table 2:** Difference-in-differences and Kernel propensity score matching difference-in-differences estimates of the effects of accreditation: 2000–2005, 2005–2008, and 2008–2014

Outcome	2000–2005		2005–2008		2008–2014	
	DiD	PSM DiD	DiD	PSM DiD	DiD	PSM DiD
Family planning						
Knowledge of side effects	16.853 (10.306)	15.777*** (3.990)	9.915** (4.275)	1.246 (3.309)	− 2.207 (2.859)	− 3.586 (3.465)
Knowledge of contraceptives	8.370 (8.253)	6.578* (3.528)	− 0.402 (4.014)	− 8.643*** (3.222)	1.622 (3.080)	0.356 (3.560)
ANC						
4 + visits	10.318 (9.359)	3.132 (3.505)	3.959 (3.742)	5.180* (2.832)	− 1.216 (2.197)	− 2.404 (2.704)
Informed of complications	12.465 (8.654)	6.430** (2.972)	10.725** (4.168)	5.454* (3.113)	3.394 (2.996)	2.046 (3.603)
Weight measurement	− 2.956 (7.004)	− 4.414 (3.012)	4.194 (2.704)	3.374* (1.981)	0.241 (1.689)	0.692 (2.086)
Blood pressure measurement	− 1.527 (6.624)	− 5.512* (2.834)	2.254 (2.698)	1.745 (2.047)	− 2.168 (1.477)	− 1.894 (1.770)
Urine sample collection	0.128 (8.645)	0.379 (3.225)	7.657** (3.796)	4.369 (2.834)	− 2.077 (2.502)	− 4.884 (3.007)
Delivery care						
Institutional delivery	15.933* (8.166)	7.043** (3.289)	− 3.661 (3.096)	− 3.224 (2.826)	0.454 (2.138)	− 0.214 (2.826)
Skilled-assisted delivery	18.138** (7.470)	11.465*** (3.154)	− 0.361 (3.004)	0.606 (2.573)	− 0.106 (1.834)	− 0.698 (2.387)
Child morbidity prevalence						
ARI	− 7.737* (4.114)	− 9.677*** (1.630)	2.299 (2.519)	1.616 (1.835)	− 0.737 (1.785)	− 1.355 (2.171)
Fever	− 8.222 (5.991)	− 10.121*** (2.178)	3.107 (2.893)	3.297 (2.169)	− 2.213 (2.107)	− 3.532 (2.478)
Diarrhea	− 5.054 (4.015)	− 4.342*** (1.515)	0.878 (2.434)	− 0.514 (1.836)	− 3.221** (1.401)	− 4.705*** (1.718)
Obs	1588	958	1531	1088	1422	1026

Each row represents a separate regression. Dependent variables are expressed in percentages. The observations are health facilities. District-level social and economic indicators as well as regional dummies are included as controls in all estimations. Clustered standard errors are reported in parentheses

*, **, and *** denote statistical significance at the 10%, 5%, and 1% levels, respectively

Using the pooled sample covering the period of 1992–2014, Table 1 shows that accreditation did not have a significant positive effect on all health outcomes except the prevalence of childhood diarrhea. This unexpected finding invited us to disentangle the observed effects of the program from each time period.

For the study comparing the years 2000 and 2005, Table 2 provides evidence that having access to an accredited facility was associated with higher likelihood of being informed of the side effects of contraceptives: the proportion of women who were informed of the side effects of the contraceptives used increased significantly by 16 percentage points (ppts) among those with access to accredited facilities, compared to women with access to non-accredited facilities. This positive effect disappeared in the subsequent periods. The proportion of women with access to accredited facilities, who were informed of other methods of contraception that could be used, increased by 7 ppts in 2005, but this effect was counterbalanced by a decrease of 9 ppts in 2008, again compared to women with access to non-accredited facilities. The effect also disappeared during the period 2008–2014.

With respect to ANC, Table 2 shows that accreditation had a limited positive effect on ANC during the period 2000–2005, specifically on being informed of signs of pregnancy complications (6 ppts); this positive effect is again observed in the period 2005–2008, but vanishes in 2008–2014. The proportion of women with access to accredited facilities, who had 4+ ANC visits or had a weight measurement during ANC visits, slightly increased significantly by 5 ppts and 3 ppts (respectively) in 2005–2008, compared to women with access to non-accredited facilities. We also observe significant negative effects of accreditation on blood pressure measurement during ANC visits in the period 2000–2005, with no significant effect in the following periods.

In terms of delivery care, institutional delivery and skilled assistance during delivery increased by more than 7 ppts and 11 ppts, respectively, among women with access to accredited facilities in the first period of analysis. Nevertheless, the estimates of both outcomes are statistically insignificant during the periods 2005–2008 and 2008–2014. In parallel, we observe that accreditation had multiple significant positive effects on child morbidity prevalence during the period 2000–2005: accreditation reduced the prevalence of childhood ARI, childhood fever, and childhood diarrhea among children with access to accredited facilities by about 10 ppts, 10 ppts, and 4 ppts, respectively, compared to children with access to non-accredited facilities. We also observe a significant positive effect of accreditation on child morbidity prevalence later during the period 2008–2014, but we do not observe any significant effects on all child morbidity prevalence outcomes during the period 2005–2008.

#### Robustness tests

We test the robustness and plausibility of our results by running several alternative checks which are discussed in Appendix C: checks and robustness extensions. Mainly, we test the parallel-trends requirement for the acceptable application of DiD; we run placebo models; we verify the common support requirement for the feasibility of the matching; we provide tests on the quality of the matching, and do several sensitivity analyses on the matching method.

## Discussion

The findings of this paper suggest that the HSRP’s accreditation process in Egypt was associated with significant improvements in child morbidity, family planning, delivery care, and—to a lesser extent—in ANC.

A comparison between the early effects of accreditation during the study period 2000–2005 versus later during the periods 2005–2008 and 2008–2014 indicates that the positive effect of the facility accreditation program faded over time. Some of the positive effects were even reversed (e.g., information on contraceptives). One explanation could be that interventions under the HSRP had been slowing down and weakening since 2005. Reviewing Egypt’s HSRP, World Bank [1] shows that the pace of implementation of the program decelerated over time. This is compounded by the extent to which facilities complied with reform rather than the rate by which facilities joined the HSRP. A plausible indicator of compliance is the accreditation score. In this regard, Grun and Ayala [41] find that although more facilities got accredited, accreditation scores were increasing until 2004 but decreased after that. Further, accreditation compliance varied across governorates. Supporting our hypothesis, using compound quality indices, El-Zanaty and Associates et al*.* [42] demonstrate that the quality of services delivered at accredited facilities improved gradually during the first four years post reform, after which it decreased and hit its lowest level after 9 years. A high level of political commitment for reform was evident in the preparation and early implementation phases, but this was not sustained, undermining the reputational gains of accreditation [43].

Our results can also be explained by the findings of Braithwaite et al. [44], who compared accreditation programs in LMICs with those in high-income countries and concluded that, regardless of country context, the sustainability of those programs is determined by continued policy support from government, stable program funding, assorted incentives promoting facilities’ participation in accreditation, and constant improvement in accreditation agency operations and program delivery. In Egypt, the HSRP has suffered from a number of institutional sustainability issues. Most critically, political support has weakened in the absence of a robust analysis of the effectiveness and rationale of the interventions proposed under the program. The relationships between parent (existing) organizations and entities newly created under the program have not been clearly delineated by the program as well [1]. On the financial front, while the initial investment costs of the HSRP were substantial, a significant fraction of these costs was financed through donor funding. The ability of the HSRP to generate its own revenues from different sources was constrained, making the program financially unsustainable in the long run [1]. In this regard, Mansour et al. [6] emphasize the lack of financial resources as a major challenge to the implementation and sustainability of accreditation programs in limited-resource settings.

The study has a number of limitations. The most notable arises from the fact that Egypt’s DHS does not track the women and children over time. To overcome this, we collapsed the data of each wave at the facility level, estimated the effects of accreditation at the facility, and constructed a longitudinal dataset. Also, our analysis on child health is limited to morbidity prevalence of common early childhood illnesses instead of treatment received because the data of Egypt DHS is patchy and incomplete on treatments. Further, being a survey, some of our calculations rely on self-reported information (number of ANC visits, information on contraceptives received, etc.), which also introduces some noise. Related to this, although the response rate is very high for each wave, there are some outcome variables with a notable higher level of missing values (contraceptive information, for instance) and we are obliged to rely on the responses available. Lastly, a small amount of randomness may be due to treatment and controls groups being defined assuming that women follow the rules and attend their nearest PHC facility, but we trust this disturbance to be very small or inexistent due to the legal obligation to do so.

## Conclusion

This paper contributes to the existing literature by investigating the effect of accreditation as a policy tool to improve quality on key patient outcomes rather than downstream process indicators, as used by most of the related literature.

We exploit six waves of the Egypt DHS to investigate the effect of HSRP’s facility accreditation programme in Egypt between 2000 and 2014 on family planning, ANC, delivery care, and child morbidity prevalence. To be able to measure the effect, we first *spatially* link women to their nearest mapped health facilities using their GPS coordinates. We then use DiD fixed-effects models and also combine DiD with Kernel PSM to correct our estimates from the potential endogeneity biases.

The results indicate that accreditation had multiple positive effects, especially on delivery care and child morbidity prevalence. The positive effects of accreditation appear to weaken over time though. Accreditation alone is not sufficient to sustain high quality of care, especially with respect to delivery care.

These results emphasize that a high, continued, level of commitment, which is a reflection of strong political will, is indispensable for the success of quality improvement interventions in LMICs in the long run. Decentralization in no way diminishes the necessity of a high level of pledge from the central government (see Braithwaite et al. [44] and Mansour et al. [6] for a detailed discussion).

Our findings encourage an enquiry into which interventions, if combined with accreditation, are associated to improved patient outcomes. There is evidence that improvements can be achieved, for example, through combining accreditation with properly monitored and well-designed payment or incentive schemes [35].

## Appendix A: the facility accreditation program

See Appendix Tables 3, 4.

**Table 3:** Score by sub-area

Quality dimension	Sub-area	Sub-area weight
Patient rights	Patient rights	2
	**Dimension total**	**2**
Patient care	General clinical areas	3
	Hypertension	3
	Diabetes	3
	ANC*	3
	Normal delivery, neonatal	3
	Postnatal care	3
	IMCI**	3
	Immunization	3
	Family planning	3
	**Dimension total**	**27**
Safety	Infection control	3
	Sterilization	3
	Employee health safety	1
	Environmental safety	2
	**Dimension total**	**9**
Support services	Emergency	2
	Laboratory	2
	Radiology	2
	Pharmacy	3
	Housekeeping	1
	Kitchen	1
	Laundry	1
	**Dimension total**	**12**
Management of information	Medical records	2
	MIS***/reporting	1
	**Dimension total**	**3**
Quality improvement program	Quality improvement program	2
	**Dimension total**	**2**
Family practice model	Prevention and screening	3
	Continuity of care	3
	Referral	3
	**Dimension total**	**9**
Management of the facility	Human resource development	1
	Management	1
	Budgeting	1
	Continuous education	1
	Provider satisfaction	1
	**Dimension total**	**5**

The scoring criteria of the accreditation standards ranges from zero to three. Scores of zero, one, two, and three denote that an accreditation standard is not met, unacceptable (partially met), acceptable (partially met), and fully met, respectively. All the scores from each activity are added to get the aggregate for the accreditation standard. The average score for each standard is calculated by dividing the aggregate scores by the frequency of activities. The scores are weighed at the sub-area score level (level one) and the overall facility score level (level two) as shown in this table

*ANC: Antenatal care. **IMCI: Integrated management of child illnesses. ***MIS: Management information system

Source: Egypt’s MOHP

**Table 4:** Overall facility score

Quality dimension	Dimension weight	% of total score
Patient rights	1	6
Patient care	5	29
Safety	3	18
Support services	2	12
Management of information	1	6
Quality improvement program	1	6
Family practice model	3	18
Management of the facility	1	6
**Total**	**17**	**100**

See notes in Table 3

Source: Egypt’s MOHP

### Box A.1: Quality dimension assessment for the different sub-areas

For the sub-area ANC, *patient care* focused on the quality of ANC at the facility, i.e., the surveyor assessed if a comprehensive history and physical examination was performed for all patients. The general physical examination should include weight measurement, height measurement, blood pressure measurement, and measurement of edema of lower limbs. The surveyor also assessed if the necessary diagnostic tests (laboratory and radiology) were performed on time to determine the diagnosis. These tests included but were not limited to blood analysis, complete urine analysis, and ultrasound according to clinical guidelines. In addition, the surveyor assessed that all treatment plans were appropriate according to clinical guidelines. For example, supplementation of iron and folic acid in first trimester was checked. The surveyor also judged the number of ANC visits according to clinical guidelines and if some educational messages were discussed with patient. For example, the physician should assist pregnant women have better knowledge and understanding of their immunization status (tetanus toxoid); the importance and the number of visits prior to delivery; alarming signs such as bleeding; and, the delivery services in the facility.

The focus of *patient care* in the sub-area IMCI was the wellbeing of children under five years of age. The surveyor assessed if a comprehensive history and physical examination was performed for all sick children according to age of child (checking for cough, diarrhea, sore throat, ear infection, and fever); if health providers explained to mothers disease classification and treatment using clear and simple language; if diagnostic tests were appropriately referred when needed; and, finally, if the facility provided appropriate prevention and treatment to all sick children according to IMCI guidelines.

The assessment of *patient care* in the sub-area family planning mainly focused on the provision and quality of counseling sessions, i.e., if a comprehensive history and physical examination was performed for all new women according to guidelines; if the facility had a good IEC system such as discussing all family planning methods and the different methods, mode of action, side effects, and costs of each.

Also, equipment had to follow international standards in accredited facilities. Thus, if needed, accreditation was accompanied by a series of interventions so that equipment would meet the expected quality standards and staff would be competent in addressing family health needs. Usually, this implied upgrading, renewing, or adding modern equipment such as sterilization ovens, delivery chairs, and dentist chairs in family health units and ensuring that there are ultrasounds and X-rays machines, and hematological and cytological labs in family health centers. To strengthen staff’s competence, equipment interventions were accompanied by a comprehensive training package for facility staff. For physicians and nurses, the package focused on family health practice. For other non-medical specialists in facilities, such as pharmacists, lab technicians, and social workers, the package focused on subject-specific training. In addition, training was a means to introduce substantial administrative changes in facilities, such as reaching out to and rostering families, and keeping medical records electronically and in family folders.

## Appendix B: data sources and descriptive statistics

See Appendix Tables 5, 6, 7, 8.

**Table 5:** Description of the Egypt DHS

	The 2000 Egypt DHS	The 2005 Egypt DHS	The 2008 Egypt DHS	The 2014 Egypt DHS
Sample selection				
Stage 1	500 primary sampling units selected (228 shiakhas/towns and 272 villages)	682 primary sampling units selected (298 shiakhas/towns and 384 villages)	610 primary sampling units selected (275 shiakhas/towns and 335 villages)	884 primary sampling units selected (481 shiakhas/towns and 445 villages before dropping North and South Sinai from the sample)
Stage 2	1000 segments from the parts in each shiakha/town and village chosen	1359 segments from the parts in each shiakha/town and village chosen	1267 segments from the parts in each shiakha/town and village chosen	1838 segments from the parts in each shiakha/town and village chosen
Stage 3	Random sample of HHs drawn	Random sample of HHs drawn	Random sample of HHs drawn	Random sample of HHs drawn
Survey coverage	17,521 HHs 15,649 women	22,807 HHs 19,565 women	19,739 HHs 16,571 women	29,471 HHs 21,903 women
Response rate	99.5%	99.5%	99.7%	99.4%

Source: The Egypt DHS, 2000, 2005, 2008, and 2014

**Table 6:** Women’s responses—raw data

Variable	Missing values	Non-missing values
Knowledge of side effects of contraceptives*	2749	36,211
Knowledge of alternative contraceptives*	19,420	19,540
4 + ANC visits**	13	37,521
Informed of complications during ANC visits**	3521	34,013
Weight measurement during ANC visits**	3514	34,020
Blood pressure measurement during ANC visits**	3515	34,019
Urine sample collection during ANC visits**	3516	34,018
Institutional delivery**	12	37,522
Skilled-assisted delivery**	19	37,515
Prevalence of childhood ARI***	5208	17,479
Prevalence of childhood fever***	5207	17,480
Prevalence of childhood diarrhea***	5207	17,480

*Conditional on reporting using any contraceptive method. **Conditional on reporting at least one birth in the last five years. ***Conditional on reporting at least one child aged 5 or under in the HH

**Table 7:** Summary statistics

Variable	Observations	Mean	Standard deviation	Minimum	Maximum
Knowledge of side effects of contraceptives	4234	39.418	28.607	0.000	100.000
Knowledge of alternative contraceptives	4133	45.983	30.315	0.000	100.000
4 + ANC visits	4598	56.235	32.182	0.000	100.000
Informed of complications during ANC visits	3520	33.598	26.091	0.000	100.000
Weight measurement during ANC visits	3522	81.778	22.777	0.000	100.000
Blood pressure measurement during ANC visits	3522	82.456	22.296	0.000	100.000
Urine sample collection during ANC visits	3522	67.863	27.034	0.000	100.000
Institutional delivery	4600	62.181	33.555	0.000	100.000
Skilled-assisted delivery	4600	72.455	29.936	0.000	100.000
Prevalence of childhood ARI	4596	13.681	15.677	0.000	100.000
Prevalence of childhood fever	4596	23.260	19.431	0.000	100.000
Prevalence of childhood diarrhea	4596	13.151	13.784	0.000	100.000
Illiteracy	15,486	33.019	9.935	0.000	56.390
Unemployment	15,486	9.491	4.287	0.000	23.550
Income dependency	15,486	0.591	0.100	0.000	0.874
Inaccessibility to electricity	15,486	1.387	4.172	0.000	69.092
Inaccessibility to potable water	15,486	4.703	7.440	0.000	95.859
Family size	15,486	4.345	0.374	2.580	6.170
HH overcrowding	15,486	1.151	0.112	0.840	1.950
Population size	15,486	31.920	16.128	0.005	117.380

**Table 8:** Two-sample *t*-test of district characteristics of accredited and non-accredited facilities

	2000–2005			2005–2008			2008–2014		
	Non-accredited	Accredited	Difference	Non-accredited	Accredited	Difference	Non-accredited	Accredited	Difference
Illiteracy	33.093	32.069	1.023 (1.138)	33.300	29.912	3.387*** (0.584)	33.351	33.107	0.243 (0.331)
Unemployment	9.548	9.061	0.488 (0.494)	9.475	9.610	− 0.135 (0.256)	9.535	9.391	0.144 (0.149)
Income dependency	0.591	0.596	− 0.005 (0.011)	0.592	0.539	0.054*** (0.006)	0.595	0.587	0.007** (0.003)
Inaccessibility to electricity	1.419	0.903	0.516 (0.487)	1.501	0.804	0.698*** (0.261)	1.946	0.711	1.235*** (0.156)
Inaccessibility to potable water	4.734	5.105	− 0.372 (0.863)	4.900	2.450	2.451*** (0.455)	5.734	3.353	2.381*** (0.268)
Family size	4.346	4.411	− 0.065 (0.043)	4.346	4.207	0.139*** (0.022)	4.358	4.304	0.054*** (0.012)
HH overcrowding	1.149	1.177	− 0.028** (0.013)	1.141	1.152	− 0.010 (0.006)	1.142	1.126	0.016*** (0.004)
Population size	31.748	29.464	2.284 (1.811)	31.482	33.685	− 2.203** (0.931)	31.521	31.176	0.345 (0.520)

Standard errors are reported in parentheses

*, **, and *** denote statistical significance at the 10%, 5%, and 1% levels, respectively

## Appendix C: checks and robustness extensions

### Parallel-trends check

The key identifying assumption of DiD is *parallel trends* in health outcomes of accredited and non-accredited health facilities in the absence of the facility accreditation program. We need to ensure that this assumption is not violated despite of two reasons. First, was accreditation targeted at health facilities already performing better (or worse) with respect to the health outcomes of interest? Second, the magnitude and even the sign of the DiD effect can be sensitive to the functional form if the outcomes’ averages for accredited and non-accredited facilities are significantly different at the baseline. The validity of the DiD estimates depends on the treated and control units being similar at the baseline. In this section, we present a number of diagnostics we ran to assess the validity of the parallel-trends assumption.

#### Pre-treatment trends in health outcomes

We use information from the 1995–2000 DHS survey waves to check for parallel trends prior to the 2000–2005 period, information from the 2000–2005 DHS survey waves to check for parallel trends prior to the 2005–2008 period, and information from the 2005–2008 DHS survey waves to check for parallel trends prior to the 2008–2014 period.

Following Mason et al. [45], we regress the change in health outcomes in the period 1995–2000 (i.e., pre-treatment slopes) on a dummy for if a facility is accredited in 2005 ‘treated’ and facility- and district-level controls^16^. Table 9

**Table 9:** Mean difference in health outcomes

Outcome	Treated		
	1995–2000	2000–2005	2005–2008
Family planning			
Knowledge of side effects	34.366 (20.842)	2.641 (6.995)	− 4.965 (5.681)
Knowledge of contraceptives		4.468 (7.134)	− 5.794 (5.497)
ANC			
4 + visits	1.367 (17.757)	− 10.662* (6.051)	0.181 (4.735)
Informed of complications		− 10.931 (6.906)	1.502 (5.752)
Weight measurement		− 5.453 (5.712)	1.541 (3.367)
Blood pressure measurement		− 1.158 (5.447)	− 2.341 (3.312)
Urine sample collection		− 7.086 (6.666)	− 0.335 (4.695)
Delivery care			
Institutional delivery	6.358 (16.107)	1.140 (5.653)	5.887 (4.315)
Skilled-assisted delivery	− 4.718 (15.898)	0.262 (5.536)	8.316** (3.686)
Child morbidity prevalence			
ARI	− 13.382 (12.820)	1.046 (4.304)	1.674 (3.017)
Fever	− 23.883 (16.119)	1.835 (4.942)	− 2.552 (3.695)
Diarrhea	− 6.025 (9.808)	2.826 (4.217)	0.416 (3.143)

Each row represents a separate specification with the dependent variable specified in the second column. Dependent variables are expressed in percentages. The observations are health facilities. The covariates are the facility characteristics, district socio-economic indicators, and regional dummies. Standard errors are reported in parentheses

*, **, and *** denote statistical significance at the 10%, 5%, and 1% levels, respectively

reports the mean changes in health outcomes between the 1995 and 2000 survey waves for facilities that are accredited versus non-accredited as of the 2005 wave, the mean changes in health outcomes between the 2000 and 2005 survey waves for facilities that are accredited versus non-accredited as of the 2008 wave, and the mean changes in our health outcomes between the 2005 and 2008 survey waves for facilities that are accredited versus non-accredited as of the 2014 wave.

The table indicates that the ‘treated’ dummy is not statistically significant for all the reported health outcomes except skilled-assisted delivery in the period 2005–2008, however the estimated effect of accreditation on this particular outcome is already insignificant and negative for the period 2008–2014 (see Table 2). As the health outcomes of accredited and non-accredited facilities had moved in tandem before the facility accreditation program started, we are confident that outcomes would have continued to move in tandem in the post-intervention period. Thus, the test for pre-trends confirms that the DiD design is valid and that the reported DiD estimators are unbiased.

#### Placebo treatment

We also run a placebo test by defining a ‘false’ lagged accreditation intervention. If the functional form of the DiD set-up is properly specified, pre-accreditation estimations should yield null results. That is, the facility accreditation program should not have a significant effect on the health outcomes of accredited facilities before being subject to accreditation. We use the data of the study period 1995–2000 to verify the results of the period 2000–2005. For the period 1995–2000, facilities that are accredited after 2000 are defined as treated and facilities that are not accredited after 2000 are defined as control. Facilities subject to additional interventions under the HSRP are removed from the dataset. We repeat the same steps to verify the results of the periods 2005–2008 and 2008–2014.

The results of the placebo test are reported in Table 10

**Table 10:** Difference-in-differences estimated effects of placebo accreditation

Outcome	1995–2000	2000–2005	2005–2008
Family planning			
Knowledge of side effects	0.556 (14.402)	5.605 (4.831)	3.668 (2.660)
Knowledge of contraceptives		9.418** (4.665)	− 3.791 (2.662)
ANC			
4 + visits	2.396 (9.444)	− 9.143** (4.276)	6.539*** (2.330)
Informed of complications		− 1.069 (3.965)	0.968 (2.490)
Weight measurement		− 10.219*** (3.930)	2.430 (1.747)
Blood pressure measurement		− 3.000 (3.906)	2.789 (1.751)
Urine sample collection		− 6.306 (4.417)	3.691 (2.420)
Delivery care			
Institutional delivery	− 1.186 (10.150)	− 2.387 (4.532)	0.978 (2.462)
Skilled-assisted delivery	− 0.225 (10.736)	− 4.243 (4.307)	3.418 (2.178)
Child morbidity prevalence			
ARI	− 5.200 (6.271)	1.223 (2.494)	0.753 (1.465)
Fever	− 9.331 (7.352)	2.886 (3.083)	0.662 (1.765)
Diarrhea	− 2.337 (4.720)	− 0.228 (2.475)	1.437 (1.488)

Each row represents a separate specification with the dependent variable specified in the second column. Dependent variables are expressed in percentages. The observations are health facilities. The covariates are the facility characteristics, district socio-economic indicators, and regional dummies. Standard errors are reported in parentheses

*, **, and *** denote statistical significance at the 10%, 5%, and 1% levels, respectively

. The treatment estimates are not significantly different from zero for all health outcomes in 2000–2005 and for the majority of health outcomes in 2005–2008. Interestingly, several health outcomes of control facilities are significantly better than that of treated facilities for the period 2000–2005. That is, differences between accredited and non-accredited facilities reported in Table 2 only emerged after the introduction of the facility accreditation program, i.e., accreditation caused the effects observed rather than the other way around.

#### Placebo outcomes

Lastly, we identify some health outcomes that, theoretically, should be unaffected by the facility accreditation program, but might be indirectly. Examples of these outcomes are modern contraceptive prevalence, ANC by skilled health personnel, tetanus immunization during pregnancy, cesarean section (C-section) rates, and under-five child mortality. If the DiD design is valid, the facility accreditation program should not have any effect on the placebo health outcomes in any study period. We re-estimate the DiD model using these outcomes and report the results in Table 11

**Table 11:** Difference-in-differences estimated effects of placebo outcomes

Outcome	2000–2005	2005–2008	2008–2014
Family planning			
Modern contraceptive prevalence	5.371 (5.681)	− 3.544 (3.055)	3.503** (1.728)
ANC			
ANC by skilled health personnel	9.358 (8.731)	0.625 (3.251)	− 3.817** (1.894)
Tetanus immunization	− 5.747 (7.729)	− 3.615 (4.247)	0.063 (2.989)
Delivery care			
C-section rate	4.663 (6.881)	− 0.017 (3.739)	3.366 (2.494)
Child health			
Under-five child mortality	− 0.289 (1.791)	1.172 (1.093)	0.027 (0.756)

Each row represents a separate specification with the dependent variable specified in the second column. Dependent variables are expressed in percentages. The observations are health facilities. The covariates are the facility characteristics, district socio-economic indicators, and regional dummies. Clustered standard errors are reported in parentheses

*, **, and *** denote statistical significance at the 10%, 5%, and 1% levels, respectively

. None of the placebo outcomes are statistically significant, which supports the validity of our DiD models.

### Common support check

A requirement for matching to be feasible is the common support or overlap condition. Thus, we check the overlap in the distribution of observable characteristics between treatment (accredited facilities) and control (non-accredited facilities) groups by visually inspecting the densities of propensity scores of both groups.

Figures ^1^, ^2^, ^3^ show that there is a large common support area or a sufficient overlap in propensity scores of accredited and non-accredited facilities to produce adequate matches for all study periods. This is expected because the number of non-accredited facilities is significantly larger than that of accredited facilities. This variation also explains why the calculated propensity scores do not exceed 0.8. In principle, if there are at least as many control units as there are treated units in the data, all the treated units can be matched, but when a small caliper is used (as in this case), the matching requires that almost all the propensity scores be less than 0.5 [46]. So, as the number of accredited versus non-accredited facilities increases in the second study period, the propensity scores increase. Plausibly, Fig. 2 shows that the control group has a higher maximum propensity score before matching, but not after matching. Figures 2, 3 also provide evidence that none of the groups has a higher maximum propensity score than the other after matching.

### Quality of matching

To check the extent to which observable characteristics are balanced in the matched sample, we perform the balancing *t*-test with the weighed covariates. Specifically, we use the balancing two-sample t-test of the difference in means of covariates across matched samples of facilities. Our covariates of interest are the ones used earlier to match treated and control health facilities. The results of the t-test are reported in Table 12

**Table 12:** Difference in mean district characteristics between accredited and non-accredited facilities

	2000–2005			2005–2008			2008–2014		
	Non-accredited	Accredited	Difference	Non-accredited	Accredited	Difference	Non-accredited	Accredited	Difference
Illiteracy	30.952	30.141	− 0.811	29.142	28.819	− 0.323	30.442	30.972	0.530
Unemployment	9.651	8.982	− 0.669***	10.126	9.83	− 0.296	10.105	9.858	− 0.247
Income dependency	0.571	0.581	0.010	0.535	0.526	− 0.009	0.561	0.563	0.001
Inaccessibility to electricity	1.188	0.716	− 0.472**	0.663	0.645	− 0.018	0.714	0.684	− 0.030
Inaccessibility to potable water	3.971	3.890	− 0.081	2.256	2.425	0.17	2.624	2.791	0.167
Family size	4.283	4.295	0.012	4.194	4.207	0.013	4.239	4.242	0.003
HH overcrowding	1.144	1.157	0.013*	1.153	1.154	0.001	1.131	1.135	0.003

Standard errors are reported in parentheses

*, **, and *** denote statistical significance at the 10%, 5%, and 1% levels, respectively

. As the table indicates, there are no systematic differences in general at the baseline in the means of observed characteristics between accredited and non-accredited facilities. That is, matching on the propensity score is successful.

### Sensitivity of results

We further inspect the sensitivity of our results to the type of the Kernel function, the bandwidth of the Kernel function, and the estimation method of the propensity score. To do the Kernel matching, we must first specify the type of the Kernel function. We initially use the Epanechnikov Kernel (the default type) to obtain our main results. In Tables 13, 14, 15, we compare the main results of the estimated effects reported to the results obtained based on other types of functions, specifically Gaussian, biweight, uniform, and tricube. Overall, we find that our main estimation results are not sensitive to the type of the Kernel function.

**Table 13:** Sensitivity to the type of the Kernel function, 2000–2005

Outcome	Main results	Type of function			
		Gaussian	Biweight	Uniform	Tricube
Family planning					
Knowledge of side effects	15.777*** (3.990)	15.859*** (3.994)	15.721*** (3.988)	15.890*** (3.995)	15.879*** (3.995)
Knowledge of contraceptives	6.578* (3.528)	6.636* (3.534)	6.506* (3.524)	6.606* (3.537)	6.641* (3.535)
ANC					
4 + visits	3.132 (3.505)	3.058 (3.512)	3.226 (3.501)	3.091 (3.513)	3.080 (3.512)
Informed of complications	6.430** (2.972)	6.220** (2.972)	6.635** (2.973)	6.182** (2.972)	6.235** (2.972)
Weight measurement	− 4.414 (3.012)	− 4.435 (3.018)	− 4.346 (3.007)	− 4.376 (3.021)	− 4.402 (3.019)
Blood pressure measurement	− 5.512* (2.834)	− 5.487* (2.838)	− 5.492* (2.831)	− 5.420* (2.841)	− 5.462* (2.840)
Urine sample collection	0.379 (3.225)	0.335 (3.233)	0.456 (3.220)	0.375 (3.236)	0.340 (3.234)
Delivery care					
Institutional delivery	7.043** (3.289)	6.991** (3.294)	7.086** (3.285)	6.965** (3.294)	6.991** (3.293)
Skilled-assisted delivery	11.465*** (3.154)	11.502*** (3.159)	11.436*** (3.150)	11.526*** (3.160)	11.507*** (3.159)
Child morbidity prevalence					
ARI	− 9.677*** (1.630)	− 9.612*** (1.634)	− 9.727*** (1.627)	− 9.590*** (1.635)	− 9.608*** (1.633)
Fever	− 10.121*** (2.178)	− 10.079*** (2.178)	− 10.160*** (2.178)	− 10.067*** (2.179)	− 10.067*** (2.179)
Diarrhea	− 4.342** *(1.515)	− 4.232*** (1.517)	− 4.439*** (1.515)	− 4.176*** (1.517)	− 4.216*** (1.517)

Each row represents a separate specification with the dependent variable specified in the second column. Dependent variables are expressed in percentages. The observations are health facilities. Standard errors are reported in parentheses

*, **, and *** denote statistical significance at the 10%, 5%, and 1% levels, respectively

**Table 14:** Sensitivity to the type of the Kernel function, 2005–2008

Outcome	Main results	Type of function			
		Gaussian	Biweight	Uniform	Tricube
Family planning					
Knowledge of side effects	1.246 (3.309)	5.312 (3.405)	1.394 (3.298)	2.106 (3.325)	1.312 (3.325)
Knowledge of contraceptives	− 8.643*** (3.222)	− 5.029 (3.325)	− 8.721*** (3.211)	− 7.860** (3.234)	− 8.356*** (3.234)
ANC					
4 + visits	5.180* (2.832)	5.264* (2.972)	5.153* (2.811)	4.806* (2.867)	5.093* (2.856)
Informed of complications	5.454* (3.113)	6.557** (3.173)	5.305* (3.103)	5.889* (3.136)	5.734* (3.128)
Weight measurement	3.374* (1.981)	4.496** (2.077)	3.374* (1.971)	3.613* (2.002)	3.421* (1.993)
Blood pressure measurement	1.745 (2.047)	2.721 (2.145)	1.731 (2.038)	1.869 (2.071)	1.766 (2.059)
Urine sample collection	4.369 (2.834)	6.270** (2.919)	4.278 (2.821)	4.443 (2.857)	4.381 (2.850)
Delivery care					
Institutional delivery	− 3.224 (2.826)	− 3.663 (2.986)	− 3.286 (2.802)	− 3.498 (2.855)	− 3.236 (2.852)
Skilled-assisted delivery	0.606 (2.573)	0.329 (2.733)	0.599 (2.551)	0.474 (2.602)	0.588 (2.597)
Child morbidity prevalence					
ARI	1.616 (1.835)	1.784 (1.883)	1.615 (1.833)	1.369 (1.849)	1.589 (1.842)
Fever	3.297 (2.169)	2.816 (2.229)	3.268 (2.163)	3.197 (2.178)	3.298 (2.176)
Diarrhea	− 0.514 (1.836)	− 0.577 (1.924)	− 0.495 (1.824)	− 0.449 (1.857)	− 0.546 (1.850)

Each row represents a separate regression. Dependent variables are expressed in percentages. The observations are health facilities. Standard errors are reported in parentheses

*, **, and *** denote statistical significance at the 10%, 5%, and 1% levels, respectively

**Table 15:** Sensitivity to the type of the Kernel function, 2008–2014

Outcome	Main results	Type of function			
		Gaussian	Biweight	Uniform	Tricube
Family planning					
Knowledge of side effects	− 3.586 (3.465)	− 3.502 (3.400)	− 3.654 (3.461)	− 3.565 (3.471)	− 3.523 (3.468)
Knowledge of contraceptives	0.356 (3.560)	− 0.031 (3.487)	0.492 (3.558)	0.118 (3.561)	0.200 (3.560)
ANC					
4 + visits	− 2.404 (2.704)	− 2.217 (2.646)	− 2.422 (2.699)	− 2.400 (2.713)	− 2.365 (2.708)
Informed of complications	2.046 (3.603)	2.375 (3.528)	1.866 (3.603)	2.185 (3.604)	2.259 (3.602)
Weight measurement	0.692 (2.086)	1.019 (2.046)	0.555 (2.083)	1.010 (2.089)	0.885 (2.088)
Blood pressure measurement	− 1.894 (1.770)	− 1.599 (1.724)	− 1.959 (1.768)	− 1.764 (1.771)	− 1.787 (1.771)
Urine sample collection	− 4.884 (3.007)	− 4.667 (2.959)	− 4.897 (3.004)	− 4.764 (3.012)	− 4.822 (3.008)
Delivery care					
Institutional delivery	− 0.214 (2.826)	0.489 (2.742)	− 0.491 (2.826)	0.203 (2.828)	0.094 (2.824)
Skilled-assisted delivery	− 0.698 (2.387)	− 0.218 (2.322)	− 0.881 (2.386)	− 0.440 (2.390)	− 0.485 (2.387)
Child morbidity prevalence					
ARI	− 1.355 (2.171)	− 0.734 (2.119)	− 1.447 (2.170)	− 1.223 (2.170)	− 1.260 (2.171)
Fever	− 3.532 (2.478)	− 2.851 (2.426)	− 3.771 (2.474)	− 3.080 (2.485)	− 3.233 (2.483)
Diarrhea	− 4.705*** (1.718)	− 3.778** (1.661)	− 4.850*** (1.721)	− 4.361** (1.710)	− 4.498*** (1.713)

Each row represents a separate regression. Dependent variables are expressed in percentages. The observations are health facilities. Standard errors are reported in parentheses

*, **, and *** denote statistical significance at the 10%, 5%, and 1% levels, respectively

To do the Kernel matching, we must also specify the bandwidth of the Kernel function. The choice of bandwidth implies a trade-off between bias and efficiency. On the one hand, a small bandwidth decreases the bias of estimates as we use the most similar observations to construct the counterfactual. The characteristics of these facilities are, in general, very similar. However, a small bandwidth decreases the efficiency of estimates as we ignore a lot of information from the sample. The fact that many control facilities are not used for the estimation implies an increase in the imprecision of estimates caused by a higher variance. On the other hand, a large bandwidth increases both the bias and efficiency of estimates. The bandwidth choice is, therefore, a compromise between a small variance and an unbiased estimate of the true density function. This choice is more important in practice than the choice of the type of the Kernel function (e.g., [47, 48].

The default bandwidth of the Kernel function initially used to obtain our main results is 0.06. Alternative bandwidths are tried (bandwidths = 0.05 and 0.1). Table 16

**Table 16:** Sensitivity to the bandwidth of the Kernel function

Outcome	2000–2005			2005–2008			2008–2014		
	Main results	Bandwidth		Main results	Bandwidth		Main results	Bandwidth	
		0.05	0.1		0.05	0.1		0.05	0.1
Family planning									
Knowledge of side effects	15.777*** (3.990)	15.777*** (3.989)	15.869*** (3.995)	1.246 (3.309)	1.868 (3.285)	3.766 (3.380)	− 3.586 (3.465)	− 3.683 (3.457)	− 3.655 (3.404)
Knowledge of contraceptives	6.578* (3.528)	6.393* (3.524)	6.640* (3.535)	− 8.643*** (3.222)	− 8.546*** (3.197)	− 6.504** (3.291)	0.356 (3.560)	0.689 (3.555)	− 0.074 (3.491)
ANC									
4 + visits	3.132 (3.505)	3.375 (3.500)	3.051 (3.512)	5.180* (2.832)	4.985* (2.789)	4.902* (2.927)	− 2.404 (2.704)	− 2.425 (2.694)	− 2.349 (2.654)
Informed of complications	6.430** (2.972)	6.872** (2.974)	6.203** (2.972)	5.454* (3.113)	5.142* (3.094)	5.977* (3.157)	2.046 (3.603)	1.612 (3.604)	2.164 (3.533)
Weight measurement	− 4.414 (3.012)	− 4.089 (3.008)	− 4.431 (3.019)	3.374* (1.981)	3.381* (1.960)	4.128** (2.047)	0.692 (2.086)	0.464 (2.080)	0.847 (2.049)
Blood pressure measurement	− 5.512* (2.834)	− 5.321* (2.835)	− 5.481* (2.839)	1.745 (2.047)	1.712 (2.029)	2.288 (2.114)	− 1.894 (1.770)	− 1.998 (1.764)	− 1.731 (1.730)
Urine sample collection	0.379 (3.225)	0.587 (3.222)	0.331 (3.234)	4.369 (2.834)	4.007 (2.806)	5.394* (2.899)	− 4.884 (3.007)	− 4.834 (3.001)	− 4.812 (2.958)
Delivery care									
Institutional delivery	7.043** (3.289)	7.079** (3.282)	6.984** (3.294)	− 3.224 (2.826)	− 3.451 (2.772)	− 3.666 (2.933)	− 0.214 (2.826)	− 0.819 (2.825)	0.417 (2.755)
Skilled-assisted delivery	11.465*** (3.154)	11.421*** (3.149)	11.504*** (3.160)	0.606 (2.573)	0.535 (2.525)	0.291 (2.682)	− 0.698 (2.387)	− 1.110 (2.384)	− 0.293 (2.331)
Child morbidity prevalence									
ARI	− 9.677*** (1.630)	− 9.699*** (1.624)	− 9.603*** (1.634)	1.616 (1.835)	1.620 (1.835)	1.442 (1.864)	− 1.355 (2.171)	− 1.588 (2.170)	− 0.955 (2.122)
Fever	− 10.121*** (2.178)	− 10.120*** (2.178)	− 10.072*** (2.178)	3.297 (2.169)	3.199 (2.157)	2.883 (2.209)	− 3.532 (2.478)	− 4.062 (2.470)	− 2.984 (2.430)
Diarrhea	− 4.342*** (1.515)	− 4.449*** (1.515)	− 4.219*** (1.517)	− 0.514 (1.836)	− 0.502 (1.812)	− 0.523 (1.895)	− 4.705*** (1.718)	− 5.020*** (1.722)	− 3.974** (1.667)

Each row represents a separate regression. Dependent variables are expressed in percentages. The observations are health facilities. Standard errors are reported in parentheses

*, **, and *** denote statistical significance at the 10%, 5%, and 1% levels, respectively

shows our main results of the estimated effects using different bandwidths. We find that our main results are not sensitive in general to the bandwidth parameter.

The estimation of propensity scores depends on a parametric specification (commonly logit or probit), which affects the quality of matching and, consequently, the results. As for the benchmark we use a probit model, we test the results when we use a logit model instead and then re-run the PSM DiD models. The results of this exercise are reported in Table 17

**Table 17:** Sensitivity to the estimation method of the propensity score

Outcome	2000–2005		2005–2008		2008–2014	
	Probit	Logit	Probit	Logit	Probit	Logit
Family planning						
Knowledge of side effects	15.777*** (3.990)	15.758*** (3.994)	1.246 (3.309)	2.180 (3.310)	− 3.586 (3.465)	− 3.540 (3.462)
Knowledge of contraceptives	6.578* (3.528)	6.513* (3.531)	− 8.643*** (3.222)	− 7.883** (3.226)	0.356 (3.560)	0.466 (3.558)
ANC						
4 + visits	3.132 (3.505)	3.128 (3.506)	5.180* (2.832)	4.912* (2.836)	− 2.404 (2.704)	− 2.451 (2.701)
Informed of complications	6.430** (2.972)	6.520** (2.973)	5.454* (3.113)	5.467* (3.108)	2.046 (3.603)	1.899 (3.600)
Weight measurement	− 4.414 (3.012)	− 4.297 (3.014)	3.374* (1.981)	3.388* (1.987)	0.692 (2.086)	0.638 (2.085)
Blood pressure measurement	− 5.512* (2.834)	− 5.435* (2.836)	1.745 (2.047)	1.832 (2.052)	− 1.894 (1.770)	− 1.902 (1.771)
Urine sample collection	0.379 (3.225)	0.394 (3.227)	4.369 (2.834)	4.401 (2.836)	− 4.884 (3.007)	− 4.882 (3.005)
Delivery care						
Institutional delivery	7.043** (3.289)	6.948** (3.288)	− 3.224 (2.826)	− 3.558 (2.829)	− 0.214 (2.826)	− 0.268 (2.827)
Skilled-assisted delivery	11.465*** (3.154)	11.395*** (3.154)	0.606 (2.573)	0.457 (2.584)	− 0.698 (2.387)	− 0.741 (2.388)
Child morbidity prevalence						
ARI	− 9.677*** (1.630)	− 9.631*** (1.629)	1.616 (1.835)	1.351 (1.832)	− 1.355 (2.171)	− 1.479 (2.168)
Fever	− 10.121*** (2.178)	− 10.015*** (2.179)	3.297 (2.169)	3.055 (2.168)	− 3.532 (2.478)	− 3.674 (2.475)
Diarrhea	− 4.342*** (1.515)	− 4.283*** (1.516)	− 0.514 (1.836)	− 0.474 (1.840)	− 4.705*** (1.718)	− 4.777*** (1.716)

Each row represents a separate regression. Dependent variables are expressed in percentages. The observations are health facilities. Standard errors are reported in parentheses

*, **, and *** denote statistical significance at the 10%, 5%, and 1% levels, respectively

. We find that the estimates for both methods of estimation match for most outcomes.

The previous robustness checks rule out an existing trend that could challenge the PSM DiD identifying assumptions. The robustness checks also provide evidence that our main estimation results are not sensitive in general to alternative types of the Kernel function, bandwidths of the Kernel function, and estimation methods of the propensity score.

## Footnotes

1 A comprehensive description of the data and survey methodology is provided online at: https://dhsprogram.com/. Table 5 in Appendix B: data sources and descriptive statistics summarizes the DHS’ sample selection, survey coverage, and response rates of the most recent four waves of the survey.

2 Table 6 in Appendix B: data sources and descriptive statistics lists the number of observations and missing values in the raw data of the Egypt DHS’ survey waves of 2000, 2005, 2008, and 2014.

3 To do so, we divide the number of women starting a new method of contraception within the 5 years preceding the survey *having been informed of its side effects/problems* by the number of all women who started a new method of contraception within the same period.

4 We divide the number of women starting a new method of contraception within the 5 years preceding the survey *having been informed about other available contraceptives prior to start* by the number of women who started the use of new modern contraceptive method within the same period.

5 An outcome of an ANC component, for instance being informed of signs of pregnancy complications, is calculated as the number of women who received ANC for their last birth within the 5 years preceding the survey *who were informed of signs of pregnancy complications* divided by the number of women with a birth within the same period who received ANC for their last birth.

6 We divide the numbers of women *who received at least four ANC visits for their last birth* within the last 5 years preceding the survey by the number of women with a birth within the same period.

7 The World Health Organization recommends that a woman receives at least four antenatal visits during a normal pregnancy to ensure that antenatal complications are detected and controlled at the earliest stage.

8 This is calculated by dividing the *number of live births in an institutional setting* within the 5 years preceding the survey by the number of all live births within the same period.

9 This is calculated by dividing the *number of live births assisted by medical provider* (doctor or nurse/midwife) during delivery within the 5 years preceding the survey by the number of live births within the same period.

10 Women who give birth at a health facility are more likely to receive proper medical attention and care during delivery, as do their infants.

11 These outcomes are calculated by dividing the number of children ill with a cough accompanied by short and rapid breathing (for ARI), or ill with a fever (for fever), or ill with diarrhea (for diarrhea) at any time during the two weeks preceding the interview, by the number of all children under five years of age.

12 Mortality due to ARI accounted for 16% globally of the total deaths among under-five children in 2015.

13 We are not able to calculate indicators of child morbidity treatment as observations in our sample are not statistically sufficient.

14 The mixture of PSM DiD was first proposed by Heckman et al. [49]. PSM DiD estimates are superior to the conventional DiD estimates as no functional form restrictions are imposed when estimating the conditional expectation of the outcome variable using PSM DiD.

15 Using weights: $\omega \left(i,j\right)=\frac{K(\frac{{P}_{j}-{P}_{i}}{{a}_{n}})}{{\sum }_{K\in C}K(\frac{{P}_{k}-{P}_{i}}{{a}_{n}})},$ where *a* is the bandwidth parameter; *K* is the Kernel function and *P*_*i*_ and *P*_*j*_ are the propensity scores for treated and control facilities.
